# Ranking Adverse Drug Reactions With Crowdsourcing

**DOI:** 10.2196/jmir.3962

**Published:** 2015-03-23

**Authors:** Assaf Gottlieb, Robert Hoehndorf, Michel Dumontier, Russ B Altman

**Affiliations:** ^1^Department of GeneticsStanford UniversityStanford, CAUnited States; ^2^Computer, Electrical and Mathematical Sciences & Engineering DivisionKing Abdullah University of Science and TechnologyThuwalSaudi Arabia; ^3^Stanford Center for Biomedical Informatics ResearchStanford UniversityStanford, CAUnited States; ^4^Departments of Genetics and BioengineeringStanford UniversityStanford, CAUnited States

**Keywords:** pharmacovigilance, adverse drug reactions, drug side effects, crowdsourcing, patient-centered care, alert fatigue

## Abstract

**Background:**

There is no publicly available resource that provides the relative severity of adverse drug reactions (ADRs). Such a resource would be useful for several applications, including assessment of the risks and benefits of drugs and improvement of patient-centered care. It could also be used to triage predictions of drug adverse events.

**Objective:**

The intent of the study was to rank ADRs according to severity.

**Methods:**

We used Internet-based crowdsourcing to rank ADRs according to severity. We assigned 126,512 pairwise comparisons of ADRs to 2589 Amazon Mechanical Turk workers and used these comparisons to rank order 2929 ADRs.

**Results:**

There is good correlation (rho=.53) between the mortality rates associated with ADRs and their rank. Our ranking highlights severe drug-ADR predictions, such as cardiovascular ADRs for raloxifene and celecoxib. It also triages genes associated with severe ADRs such as epidermal growth-factor receptor (EGFR), associated with glioblastoma multiforme, and SCN1A, associated with epilepsy.

**Conclusions:**

ADR ranking lays a first stepping stone in personalized drug risk assessment. Ranking of ADRs using crowdsourcing may have useful clinical and financial implications, and should be further investigated in the context of health care decision making.

##  Introduction

Pharmacovigilance plays a crucial role in the continuing evaluation of drug safety. Adverse drug reactions (ADRs) contribute to excess length of hospitalization time, extra medical costs, and attributable mortality [1,2]. Thus, assessment of the impact of ADRs on drug risk-benefit assessment has gained significant interest in recent years as several risk-benefit methodologies have been suggested for assessing drug safety and efficacy [3,4]. Two factors are essential for risk assessment: the prevalence of the ADR in the population (ie, frequency) and the severity of the ADR in terms of medical (morbidity and mortality) or financial consequences. Risk estimates focus mainly on ADR frequency, as there is no publicly available resource that provides estimates of relative severity of ADRs. Thus, these methods either handle a single ADR at a time [3] or assign equal weights for all the drug ADRs [5]. However, not all ADRs are of equal interest: life-threatening ADRs require more attention, while minor ADRs may not. Although a few severe life-threatening ADRs are well recognized, including liver failure, cardiac arrest, and others, there is presumably a gradation of severity from these down to the most benign. Of course, patients’ subjective perception of the severity of an ADR varies widely, and so a ranking of ADRs is fundamentally a personal activity when it comes to individual patient decisions. Nonetheless, a ranking of ADRs based on perceived severity is a useful starting point for risk-benefit assessment and for patient-centered care, and is the focus of this paper.

Ranking large sets of ADRs is challenging; theoretical analyses have provided a framework for such evaluations [6,7]. Tallarida et al asked 53 physicians to assign weights to seven severity classes, but their study contained ADRs specific to only two drug classes (treating hypertension and rheumatoid arthritis), and thus has limited general utility. In a subsequent work, the authors showed consistent ranking between the 53 physicians and 56 non-professional rankers. Encouraged by this result, we sought to crowdsource rankers to obtain a more comprehensive ADR ranking. In order to accomplish this, we divided this complex task into simpler microtasks (pairwise comparisons), well-suited to a crowdsourcing platform, such as the Amazon Mechanical Turk (MTurk). MTurk is a crowdsourcing microtask platform (microtasking refers to tasks that are divided into multiple smaller subtasks) that allows human workers to perform tasks in return for payment (see Methods for extended description). Previous evaluations have shown that MTurk can be as reliable as traditional survey methods, and that the use of control validation questions can markedly improve reliability and reduce variability [8]. To the best of our knowledge, crowdsourcing has not been used for pharmacovigilance applications yet.

Our goal was to rank the ADRs by severity from a population (non-expert, non-clinician) perspective. We ranked a list of 2929 ADRs by assigning 126,512 ADR pairwise comparisons to 2589 individuals and processing the comparisons with an optimization algorithm to rank the ADR severities.

ADRs are reported in drug labels following clinical trials. Additional drug-ADR associations can be inferred, both empirically, through reporting systems such as the US Food and Drug Administration (FDA) Adverse Events Reporting System (AERS), or based on computational predictions (using drug similarity [9], genetic overlap [10], and pathway analysis [11]), followed by pharmacoepidemiological studies to confirm these predictions. These associations are numerous: the average FDA label lists 100 ADRs and some prediction work suggests that the number of ADRs may be doubled. A severity ranking would be useful to triage ADR predictions for further investigations. In the discussion, we list three additional uses for our ranked list, including reduction of “alert fatigue”, whereby alerts are ignored [12], identification of ADRs that suggest a larger component of patient preference, and association of individual genes with ADR severity. We make the raw data upon which our analysis is based and the resulting ADR ranking publicly available.

## Methods

### Data

ADRs were retrieved from the “SIDER2” side effect database (October 2012 version, listing total of 4192 ADRs) [13]. Predicted ADRs-drug associations mined from the FDA Legacy AERS were retrieved from [14] (“OFFSIDES” off-label side effect database). Gene-ADR associations assembled from literature were retrieved from [15] and predicted gene-ADR associations based on inferred pharmacodynamics pathways were retrieved from [11] (DrugRouter). ADRs from SIDER2, AERS, and OFFSIDES are coded using the medical dictionary for regulatory activities (MedDRA) terminology [16].

The AERS data files were downloaded from the FDA website [17]. The data covered the interval first quarter, 2004, through third quarter, 2012. All files were imported into an SQLite database and fields were checked against a list of allowed values drawn from documentation supplied by the FDA in the download. Three formatting errors were corrected manually. Individual safety reports were aggregated into cases, removing duplicate reports per case (follow-ups). The top 100 prescribed drugs in 2013 were retrieved from [18].

Semantic similarity between ADRs was computed using the Human Phenotype Ontology (HPO) [19], downloaded on May 30, 2014, using the Semantic Measures Library v0.8 [20].

### What is Amazon Mechanical Turk (MTurk)?

MTurk is a platform for task creation, labor recruitment, and compensation. “Requesters” create and publish “human intelligence tasks” (HITs) and “workers” complete these tasks. The tasks are ones that can be completed using a computer and typically require a short time to complete, with a corresponding small compensation. Prior to posting a task, the requester sets the compensation amount (Amazon charges an additional 10% commission). Workers can browse and choose from available tasks and are paid upon successful completion of each task. Requesters can also reject subpar work. In this case, the rejected workers do not receive payment and it also negatively affects the worker record as requesters may limit their tasks to workers with low rejection rates.

### Ranking 2929 Adverse Drug Reactions

**Figure 1:**
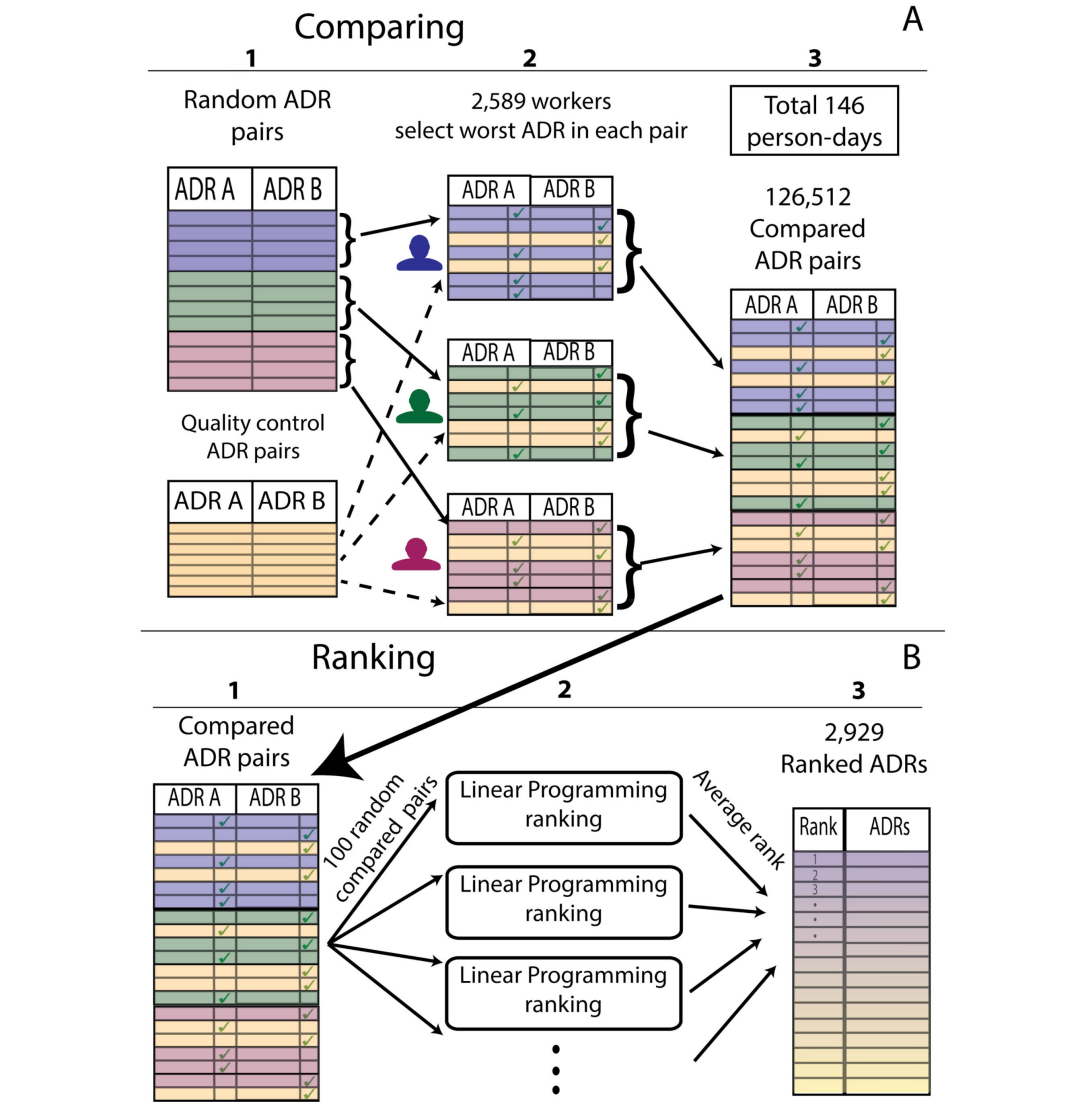
MTurk task construction (A) and ranking process (B). (A) Random list of pairwise comparisons and list of predefined quality control pairs are constructed (1). Each worker receives unique set of 7 random ADR pairs to compare and 3 quality control pairs for performance evaluation (2). Results are collected and merged (3). (B) Ranked pairs are sampled (1), sent to a linear programming task (2), and ranking of each sample merged to a global ranking (3).

We retrieved a set of 2929 common ADRs (expressed in the MedDRA terminology) from drug labels, as represented in the SIDER2 database [13]. All 2929 ADRs were also reported in the FDA AERS, which collects ADR reports from clinicians, patients, and pharmaceutical companies. We used the Amazon MTurk crowdsourcing platform to assign 126,512 ADR comparison tasks to 2589 individual rankers (“workers” in MTurk terminology) (Figure 1 A). Each worker was presented with up to 15 sets of 10 pairwise ADR comparisons and was requested to select, for each pair, which ADR is more severe. The user interface provided clickable links to Google queries with the ADR name in order to help workers learn about ADRs expressed in medical terminology with which they were not familiar (Multimedia Appendix 1 displays an example set of comparisons presented to workers).

The workers were required to possess satisfactory task completion records, rejected in less than 5% of past tasks (95% approval rate), and be located in the United States, as a proxy to English proficiency. In order to identify reliable workers, each worker task of 10 pairs included three pre-defined quality control pairs with expected answers and seven randomly chosen pairs. These quality control pairs were constructed by pairing all combinations from a manually selected set of severe ADRs and a set of mild ADRs.

Using the pre-defined set of quality control comparisons, we removed inconsistent workers who did not answer these appropriately, resulting in 124,513 usable pairwise comparisons (57,901 unique comparisons, multiple comparisons were made for consistency evaluation, see Multimedia Appendix 2 for raw comparisons).

In construction of the pairwise comparisons, we took the following measures in order to maximize the tested pairs and reduce as much as possible potential biases: (1) the tasks were distributed on different weekdays over a period of 1 month, and (2) using an initial crude ranking computed from the first batch of comparisons, we randomly selected the ADR pairs that were not too easy (comparing a severe and a mild ADR) or equivalent (ADRs with very close ranks), as equivalent ADRs are harder to compare and have the potential to frustrate the MTurk workers in being forced to choose.

A quality control batch of pairwise comparisons (14,645 pairs) was repeated three times to assess reproducibility. It was also constructed to maximize the number of pairs that can be tested for triangular inequality (ie, for ADRs A, B, and C, test A vs B, B vs C, and A vs C).

Each task, consisting of 10 pairwise comparisons, took 5 minutes to complete on average, yielding US $0.45 per worker (half a dollar including Amazon’s fee). The entire ranking totaled in 146 person days at a cost of US $6,300. A more detailed description and worker statistics are found in Multimedia Appendix 3.

### Ranking Adverse Drug Reactions

We formulated a linear programming scheme to compute a ranked list of the ADRs from the pairwise comparisons (illustrated in Figure 1 B). Due to time and budget constraints, we were able to sample only a small fraction (1.5%) of the nearly 4.3 million possible ADR pairs. In order to rank the ADRs based on the pairwise comparisons, we used linear programming that attempted to retain as much of the original rankings of the workers (in the minimization of the utility function) while ensuring that the ADRs obeyed the triangular inequality, that is, for each ADR triplet A, B, and C, we denote more severe as “greater than”, so if A>B and B>C then it follows that A>C. The linear programming optimization function and constraints are found in Multimedia Appendix 3, Ranking ADRs section.

The linear programming was implemented in MATLAB using IBM CPLEX package version 12.6 [21].

### Consistency of Adverse Drug Reaction Rankings

#### Overview

We estimated the consistency of pairwise comparisons using a batch of comparisons that was constructed for quality control purposes. It was repeated three times and included multiple ADR triplets that were tested for triangular relationships. Specifically, for each ADR (A), we included 10 comparisons that formed 10 testable triangular relations (ie, for ADRs B and C, we included the three comparisons A vs B, A vs C, and B vs C).

We tested the reproducibility of the ranking across the three repeated batches. Only 16% of the workers participated in more than one of the repeated batches (13% in two batches, and 3% in all three batches).

#### Adverse Drug Reaction Ranks Are Associated With Relative Deaths From Adverse Events Reporting System (AERS) Reports

We counted the number of reports associated with an ADR in the AERS and the number of reports specifying one of the six outcomes (death, disability, life-threatening, required intervention to prevent permanent impairment/damage, hospitalization, and congenital anomaly). The rate of each outcome per ADR is the number of reports with that outcome divided by the total number of reports for that ADR, including reports with non-specific outcome tagged as “other serious” (25%) and reports with no outcome specified (20%).

In order to extract the major outcomes associated with the severity ranking, we used the lasso regression method [22] with a 10-fold cross validation. Relative death rate was the leading factor (producing 4% increase over the best mean standard error).

## Results

### Ranking 2929 Adverse Drug Reactions

We ranked a set of 2929 common ADRs from the SIDER2 database [13] using workers from the Amazon MTurk crowdsourcing platform (see Figure 1 A, and Methods). We used a pre-defined set of quality control comparisons with expected answers to remove inconsistent workers. A total of 90% of workers answered correctly on all the quality control pairs. We formulated a linear programming scheme to compute a ranked list of the ADRs from these pairwise comparisons (see Figure 1 B and Methods). The most severe ADRs were cardiac arrest and metastatic bone cancer and the least severe were euphoric or elevated mood. We list the 20 most and least severe ADRs in Table 1 and the full ranked list in Multimedia Appendix 4.

**Table 1:** Top- and bottom-ranked ADRs.

Rank	Top-ranked severe ADRs	Rank	Bottom-ranked mild ADRs
1	Cardiac arrest	2910	Growth of eyelashes
2	Bone cancer metastatic	2911	Eye rolling
3	Left ventricular failure	2912	Night sweats
4	HIV infection^a^	2913	Chapped lips
5	Anal cancer	2914	Nasal congestion
6	Lung cancer metastatic	2915	Agitation
7	Hemorrhage intracranial	2916	Excitability
8	Chronic myeloid leukemia	2917	Breath odor
9	Coma	2918	Hair growth abnormal
10	Breast cancer	2919	Hot flush
11	Multi-organ failure	2920	Sleep talking
12	Cardiopulmonary failure	2921	Blister
13	Cardiac death	2922	Tongue dry
14	Chronic leukemia	2923	Moaning
15	Cardio-respiratory arrest	2924	Discomfort
16	Pulmonary embolism	2925	Decreased appetite
17	Completed suicide	2926	Dry mouth
18	Metastatic renal cell carcinoma	2927	Early morning awakening
19	Hepatic angiosarcoma	2928	Euphoric mood
20	Anaplastic thyroid cancer	2929	Elevated mood

^a^HIV: Human immunodeficiency virus infection, while not caused by a drug, is associated in with several drugs in SIDER.

### Consistency of Adverse Drug Reaction Rankings

We estimated the consistency of pairwise comparisons by repeating a quality control batch of pairwise comparisons three times. The batch included multiple ADR triplets that were tested for triangular relationships. Only 10% (SD 0.3%) of these ADR triplets violated the triangular inequalities (total of 23,071-26,245 triplets in each batch repeat, variation is due to exclusion of workers judged inconsistent on pre-defined quality control pairwise comparisons).

**Figure 2:**
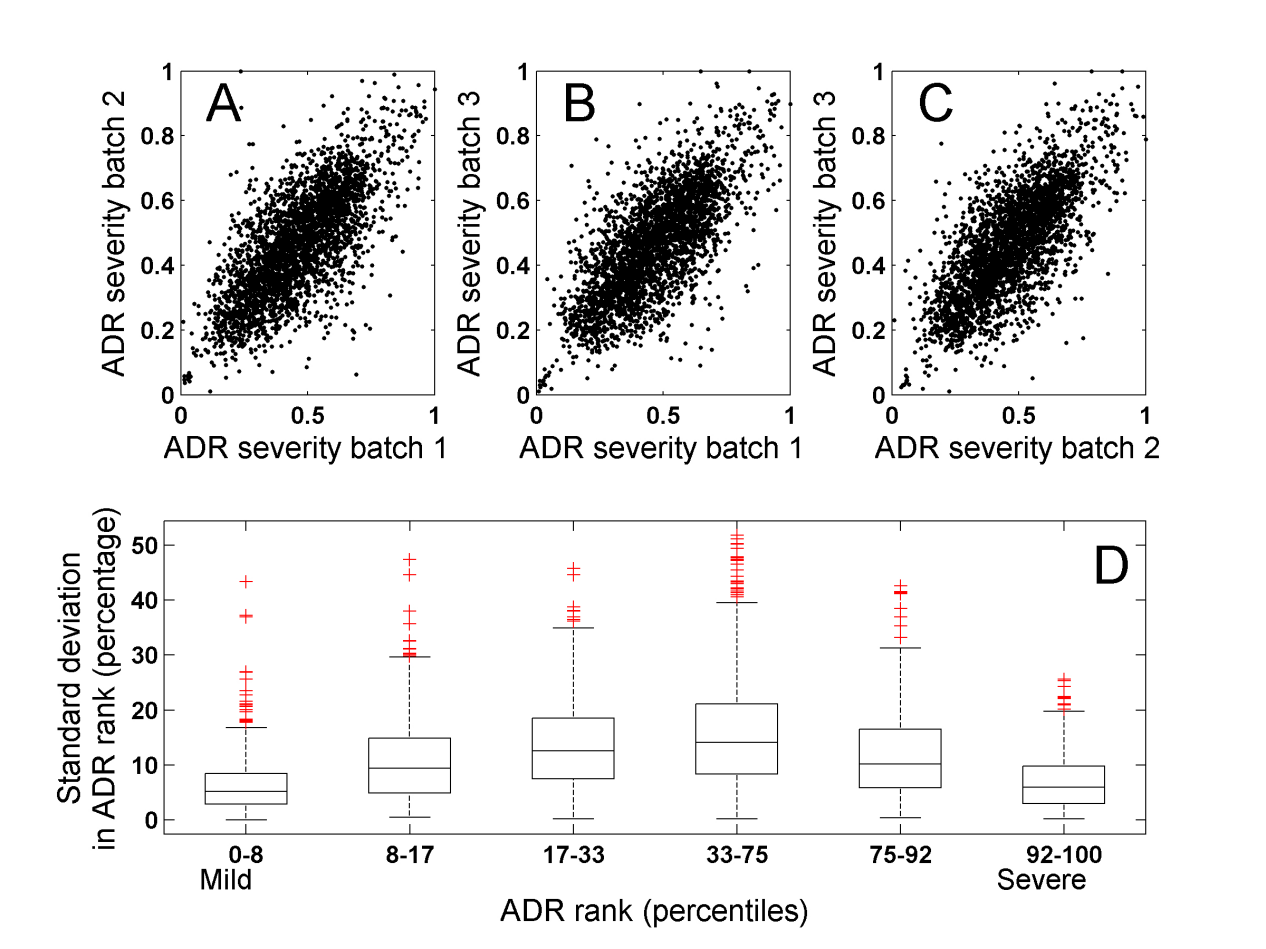
Correspondence between duplicate quality control batches. Ranking correlation between duplicate batches 1-3 (A-C) and a box-plot of the standard deviation in rank scores across the 3 batches as a function of the score (D).

We next tested the reproducibility of the ranking across the three repeated batches (see Methods). Among pairs compared by at least three different workers from these three duplicate batches, 58% had full agreement. Despite this agreement, the Spearman correlation coefficient between the ranking independently computed from the three duplicate batches was .71 (SD .009, *P*<.001) (Figure 2 A-C). Based on the standard deviation of ranks across the three repeated batches, a one-sided analysis of variance identified six robust classes of ADRs (*P*<.001) (see Figure 2 D and Multimedia Appendix 3 for details). Among the ADRs with highly variable rank, we find hemolysis, tracheooesophageal fistula, actinic keratosis, suicidal ideation, and chronic otitis media. ADRs with the least variable rank included furuncle, moaning, chapped lips, and discomfort.

Finally, ADRs sharing high semantic similarity exhibited smaller difference in their severity ranks (Pearson correlation ρ=−.94, *P*<.001) (see Multimedia Appendix 5).

### Severe Adverse Drug Reactions Are Associated With More Deaths in the FDA Adverse Events Reporting System (AERS)

**Figure 3:**
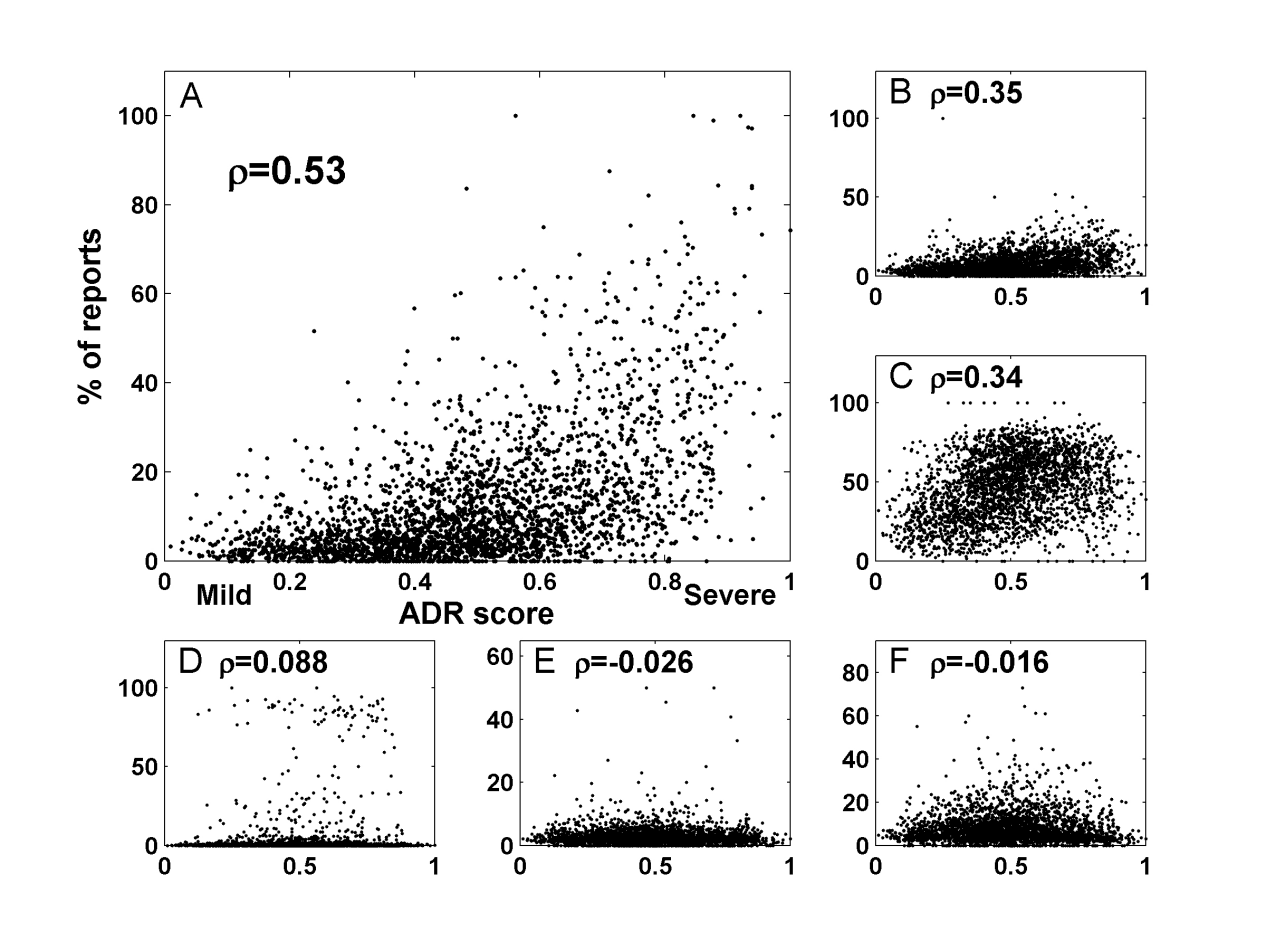
Correlation between ADR rank and outcomes. Severe ADRs tend to have significantly higher death rate (A), moderate correlation with life-threatening (B), and hospitalization (C), and negligible correlation with congenital anomaly (D), required intervention to prevent permanent impairment/damage (E), and disability (F).

**Figure 4:**
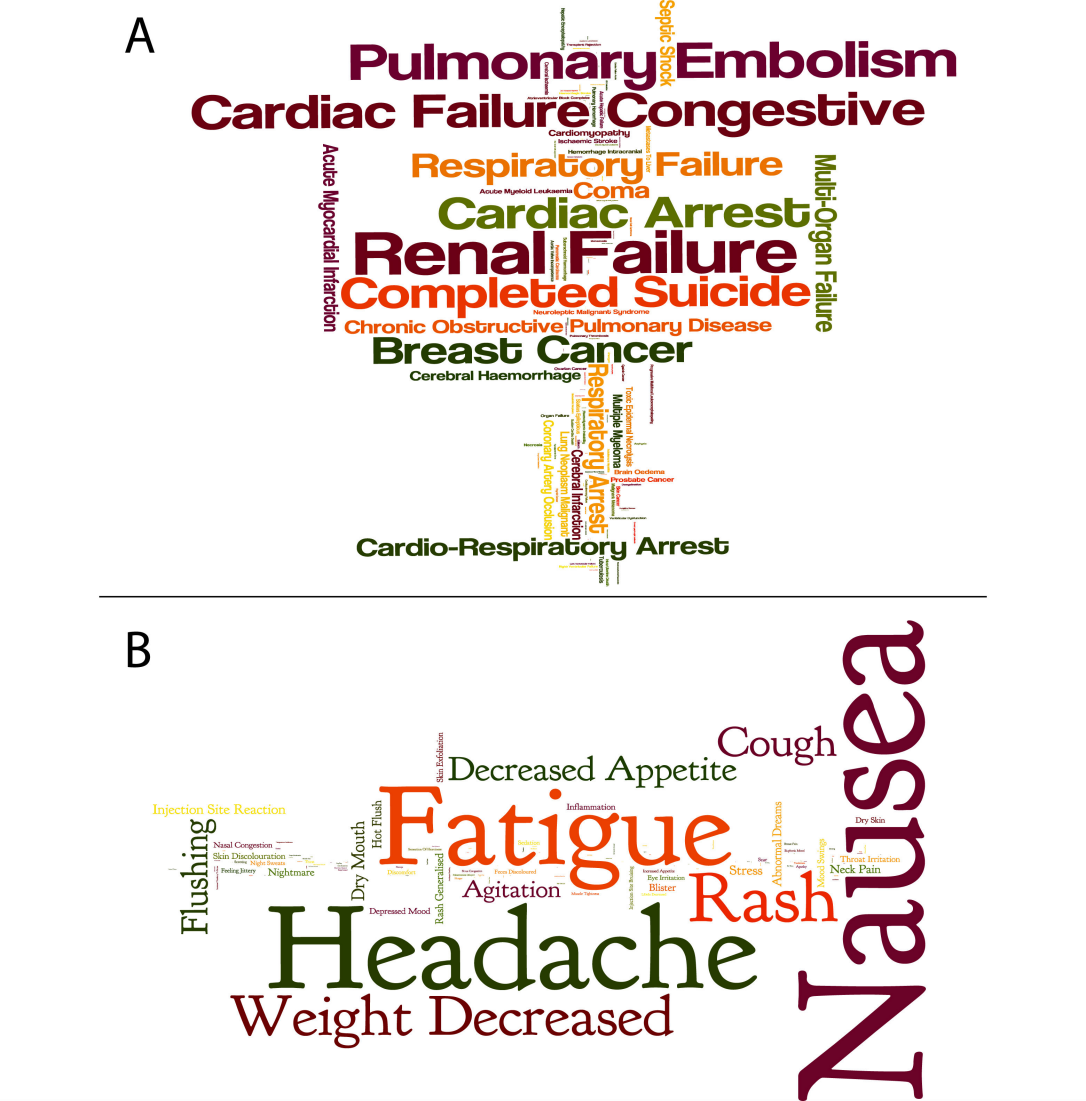
Term clouds for top 95 percentile ADRs (A) and bottom 5 percentile (B). Term size is proportionate to the relative number of reports in the FDA AERS.

AERS contains reports on adverse event submitted to the FDA. Some of the reports include a specific outcome of the ADR (55% of the reports including ADRs in our set). These specific outcomes are death, disability, life-threatening, required intervention to prevent permanent impairment/damage, hospitalization, and congenital anomaly. We found a significant correlation between the relative death rate in AERS reports (ie, the relative number of deaths out of all ADR reports) and our severity rank for the ADR (ρ=.53, *P*<.001) (Figure 3 A). Additionally, life-threatening and hospitalization outcomes were moderately correlated with our ranking (ρ=.35 and ρ=.34, respectively, *P*<.001 for both) (Figure 3 B and C). The other possible outcomes were not strongly correlated with our ADR severity (see Figure 3 D-F and also the Discussion). Death rate is also the highest contributing factor in a lasso regression [22] of the AERS outcomes percentages against the ADR score (Methods). There are exceptions that illustrate the limits of this evaluation: idiopathic pneumonia syndrome (an ADR associated with high dose carmustine chemotherapy [23]) was ranked at the 48^th^ percentile in severity but displays a high relative death rate (84%). Conversely, breast and thyroid cancers are ranked among the 99^th^ percentile of severe ADRs, but have lower than 10% reported mortality rate in the AERS. We found no significant correlation between the ADR severity ranking and patient demographic traits (age and gender) in the AERS reports. Figure 4 displays a cloud of the most and least severe ADRs, sized by their relative mentioning in the AERS system, displaying wide variation across different ADRs of similar ranking.

### Some Therapeutic Classes Have More Severe Types of Adverse Drug Reactions Than Others

Drug risk assessment is affected by the severity of its associated ADRs and by their frequency in the population. In order to evaluate the reliability of ADR frequencies, we surveyed drug labels for 65 severe and frequent drug-ADR associations, where we define severe ADRs as those ranked above the 95^th^ percentile and frequent drug-ADR association as those reported with larger than 1% frequency in the SIDER database. The frequency information in those labels was largely insufficient to estimate the marginal frequency above a control (ie, a placebo). Only two associations (3%) were compared to a control group that underwent a procedure (orchiectomy) instead of receiving a different drug. The reported frequency was significantly higher than that control (5% occurrence for congestive cardiac failure and chronic obstructive pulmonary disease after administration of zoladex, vs 1% for the control, *P*<.001). We thus disregarded frequency information and focused on the most severe ADRs, assuming they are essential to highlight until their frequencies are determined.

**Figure 5:**
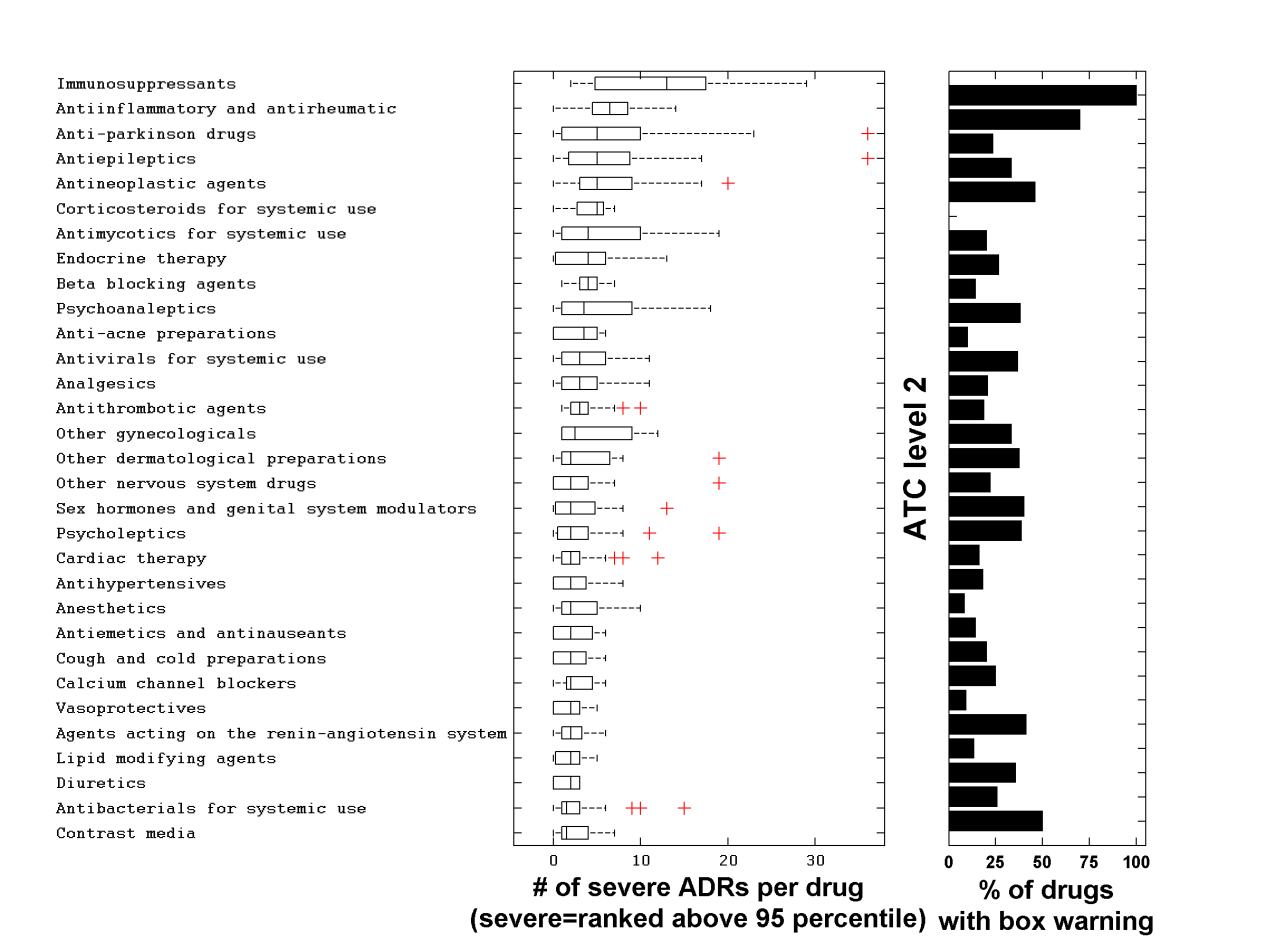
Severity of ATC classes. Box plot of ATC class severity measured by number of severe ADRs in each class (severe defined by top 95 percentile of the ranks) and percentage of drugs with black box warning in that class. Only classes that include more than 2 drugs with ADR information and have at least more than 3 severe ADRs are displayed.

We associated ADRs with a set of therapeutic drug classes by aggregating the drug-ADR associations according to therapeutic class, as defined by the second level of the drug Anatomical Therapeutic Chemical (ATC) Classification System. We counted the number of different severe ADRs per drug as mapped in SIDER. Aggregated across the ATC classes, we identified classes with high variability among drugs in terms of the number of associated severe ADRs (Figure 5). The median number of severe ADRs per therapeutic class is positively correlated to the fraction of drugs having FDA box warnings for that category (ρ=.64, *P*<.001). We highlight two classes that display high numbers of associated severe ADRs (median ≥5 severe ADRs) and large variability (SD>9). Immunosuppressants include drugs associated with only two (azathioprine) or three (leflunomide) severe ADRs as well as drugs associated with a high number of severe ADRs (lenalidomide associated with 29 and tacrolimus with 19). The severe ADRs that are associated with the highest number of immunosuppressants are necrosis, renal failure, and congestive cardiac failure. Anti-Parkinson drugs include drugs associated with no severe ADR (orphenadrine, biperiden, and benztropine) as well as drugs associated with multiple severe ADRs (pramipexole, associated with 36 and ropinirole, associated with 23). The most common severe ADRs among anti-Parkinson drugs are cardiac arrest, coma, renal failure, skin cancer, and cerebral ischemia.

### Ranking Can Prioritize Predicted Adverse Drug Reactions

A recent study predicted drug-ADR associations using a statistical analysis of AERS (438,801 drug-ADR pairs [14]). An experimental validation of such a large set of predictions is impractical, and yet regulators need to prioritize such associations for further investigation. Since ADR frequencies cannot be determined from the spontaneous reporting in the AERS, we highlight severe ADRs occurring in highly prescribed drugs, for which even low frequencies would have significant impact. We focused on the top 100 prescribed drugs in 2013 [18], and identified 53 drugs that have novel severe ADRs in the OFFSIDES databases (Multimedia Appendix 6). We highlight two drugs with the largest number of novel severe ADRs. First, pulmonary embolism is listed on the drug label raloxifene, an elective estrogen receptor modulator. OFFSIDES database predicts an associated ADR, pulmonary thrombosis, and additionally carotid artery and cerebral thrombosis. Second, celecoxib, a non-steroidal anti-inflammatory drug, is associated with myocardial infarction, acute coronary syndrome, and transient ischemic attack, which are a result of the predicted coronary artery occlusion and cardiomyopathy. Furthermore, the predicted cardiomyopathy often leads to the reported cardiac failure.

### Triaging Genes Associated With Adverse Drug Reactions

During drug development it is useful to identify genes and pathways that are associated with ADRs; it may be even more useful to quantitatively compare these using our severity ranking. Accordingly, we used gene-ADR associations assembled from literature [15] and used the integrated ADR severity for each gene to score its “adverse reaction risk”. Multimedia Appendix 7 lists the genes and their most severe associated ADR. Our analysis highlights previously associated genes and severe ADRs [15], as shown in Table 2. We also highlight three genes predicted by [11] to be associated with neuroleptic malignant syndrome (NMS): HTR2A, NGPR, and COMT.

**Table 2:** Genes reported to be associated with severe adverse drug reactions (ADRs) (top 10 percentiles).

Gene	ADR (Percentile)	Reference
EGFR	Glioblastoma multiforme (95)	[24]
SCN1A	Epilepsy (93)	[25]
VDR	Chronic renal failure (91)	[26]
TNF	Multiple sclerosis (91)	[27]
RYR1	Malignant hyperthermia (90)	[28]

## Discussion

### Principal Results

We ranked the severity of 2929 ADRs using a crowdsourcing platform. This ranking helps highlight drug classes based on the severity of their associated ADRs, triage predicted drug-associated ADRs for further investigation, and associate genes with a severity score based on their association with ADRs, with some implications for drug design. Although our ranking is consistent and reproducible, we cannot claim that it is optimal. A broader sampling of the potential ADR space (perhaps including professionals and patients who have experienced these effects) or a more sophisticated ranking method might improve the quality of the ranking. We include the raw pairwise comparison data (Multimedia Appendix 2) and our rankings in order to enable alternative analyses.

### Limitations

Our ranking is based on a non-expert and inexperienced understanding and interpretation of ADR severity. Our analysis includes both point events and interval events, and these were compared without (1) reference to their different time courses, or (2) variations in severity between different instances of the same ADR—the MTurk workers were simply asked to decide if one ADR was better or worse than another, integrating all considerations. The high performance on the quality control ADR pairs (marked in Multimedia Appendix 2) and the consistency of the ranking shows that they generally comprehended the medical terms (possibly through using the provided Google query links). The average completion time of a survey (comprised of 10 pairwise comparisons) was 5.33 minutes, which is higher than the average time (4 minutes) required for a biomedical scientist to complete such surveys. Last, we included only workers from the United States, but our method may be biased by other demographic traits of the workers. While we have no access to such information in our study, we estimate from other sources that the average age is 33-35 years old and 63%-72% females (19).

As mentioned above, we identified some ADRs with discordance between our estimated severity and their mortality rate in the AERS reports. There are two reasons for such discrepancies: (1) a misunderstanding by laymen of the true severity of an ADR (eg, the word “cancer” may get a high ranking, regardless of its survival statistics), and/or (2) a bias in the associated death rates in the AERS system. We are unable to distinguish these, and it is likely that both contribute, highlighting areas for potential improvement.

There is no correlation between the outcome rates of disability, required intervention to prevent permanent impairment/damage, or congenital anomaly to our ADR ranking. After manual examination of the ADRs with high rates for these three types of outcomes, we identified that for the first two, disability and “required intervention” outcomes, a lack of context caused ADRs with high rates to be classified as mild. For example, grimacing or rectal cramps are associated with more than 55% disability rate, and may be frequent disability co-occurring ADRs. Similarly for “required intervention”, light anesthesia (>42% rate) and hyposmia (>25% rate) are moderate without context. In the case of congenital anomaly, many of the anomalies are not life threatening and thus were ranked low (eg, supernumerary nipple, low set ears, or ear malformation).

Finally, we used the list of ADRs appearing in SIDER and the FDA AERS systems “as-is”. Some of the ADRs in our list may not be directly caused by drugs but are associated with drugs (eg, infections may be more frequent as a side effect of the drug, or may simply co-occur with diseases that the drug treats). We retained these ADRs, as they provide important insight regarding how individuals perceive their relative severity.

### Implications

We highlight drug therapeutic classes that display large variability between their drug members in terms of occurrences of severe ADRs, suggesting staying vigilant in regard to the effect of drug choice on ADR occurrence in patients. We also highlight genes associated with severe ADRs, which should be subject for further investigations.

Among the potential applications for a ranked list of ADRs, we suggest that mapping these ADRs to drug-drug interactions could aid in reducing “alert fatigue” stemming from too frequent alerts, which often emerge on relatively mild events. This phenomenon may cause physicians to dismiss these alerts and could possibly be attenuated if the alerts focused mostly on major adverse event [29]. Certain ADRs deviate more than others in rank, suggesting that their perceived severity is more of a personal preference (Multimedia Appendix 4). This information could identify cases where patient preferences should be weighted more strongly when making a prescribing decision.

Finally, we focused on the severity of ADRs, but ADR frequency is also crucial for assessment of drug risk. These ADR frequencies require proper control to correct for background frequencies. Carefully constructed clinical trials that allow extracting statistically significant frequencies in a rigorous way should be given high priority.

### Conclusions

We believe that our ranking of ADRs may have useful clinical and financial implications, and should be further investigated in the context of health care decision making.

## Multimedia Appendix 1

An example of a comparison presented to an MTurk worker.

## Multimedia Appendix 2

Table S1. The MTurk workers pairwise comparisons used to compute the ranking.

## Multimedia Appendix 3

Supplementary methods, figures, and Multimedia Appendix legends.

## Multimedia Appendix 4

Table S2. Ranked list of ADRs with their reported frequency.

## Multimedia Appendix 5

Correlation between ADR semantic similarity and mean difference in severity scores, computed for 793 ADRs.

## Multimedia Appendix 6

Table S3. Top prescribed drug in 2013 that have novel severe ADRs in OFFSIDES database.

## Multimedia Appendix 7

Table S4. Genes and their most severe associated ADRs.

## Abbreviations

ADR: adverse drug reaction
AERS: Adverse Events Reporting System
ATC: Anatomical Therapeutic Chemical Classification System
FDA: Food and Drug Administration
HIT: human intelligence tasks
HIV: human immunodeficiency virus
HPO: Human Phenotype Ontology
MedDRA: medical dictionary for regulatory activities
MTurk: Amazon Mechanical Turk
