# Supplementary figures and images for: Ranking Adverse Drug Reactions With Crowdsourcing
Source: J Med Internet Res. 2015 Mar 23;17(3):e80. doi: 10.2196/jmir.3962 (PMC4387295; doi:10.2196/jmir.3962)

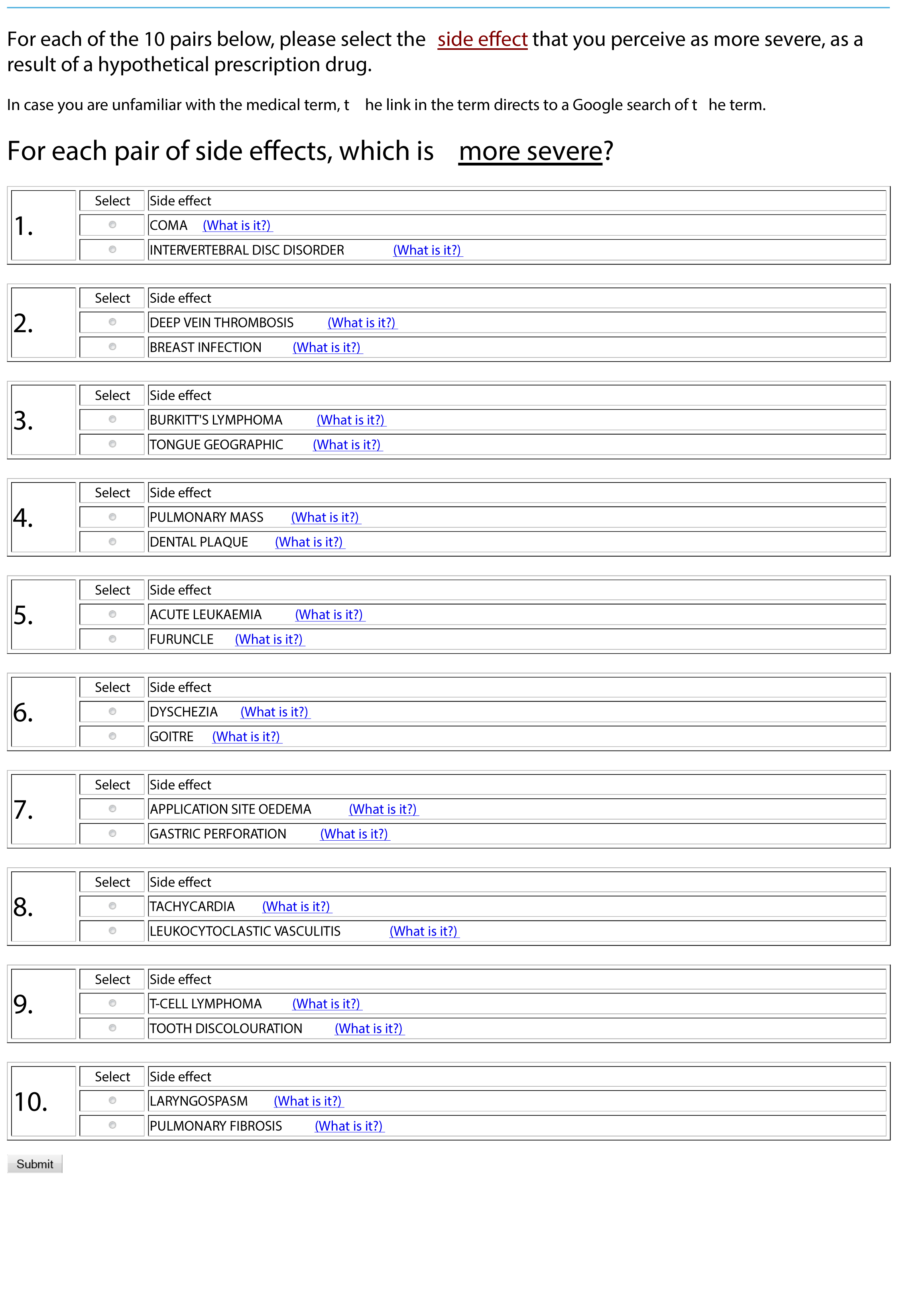

Supplement: Supplementary file 1 [file jmir_v17i3e80_app1.png]

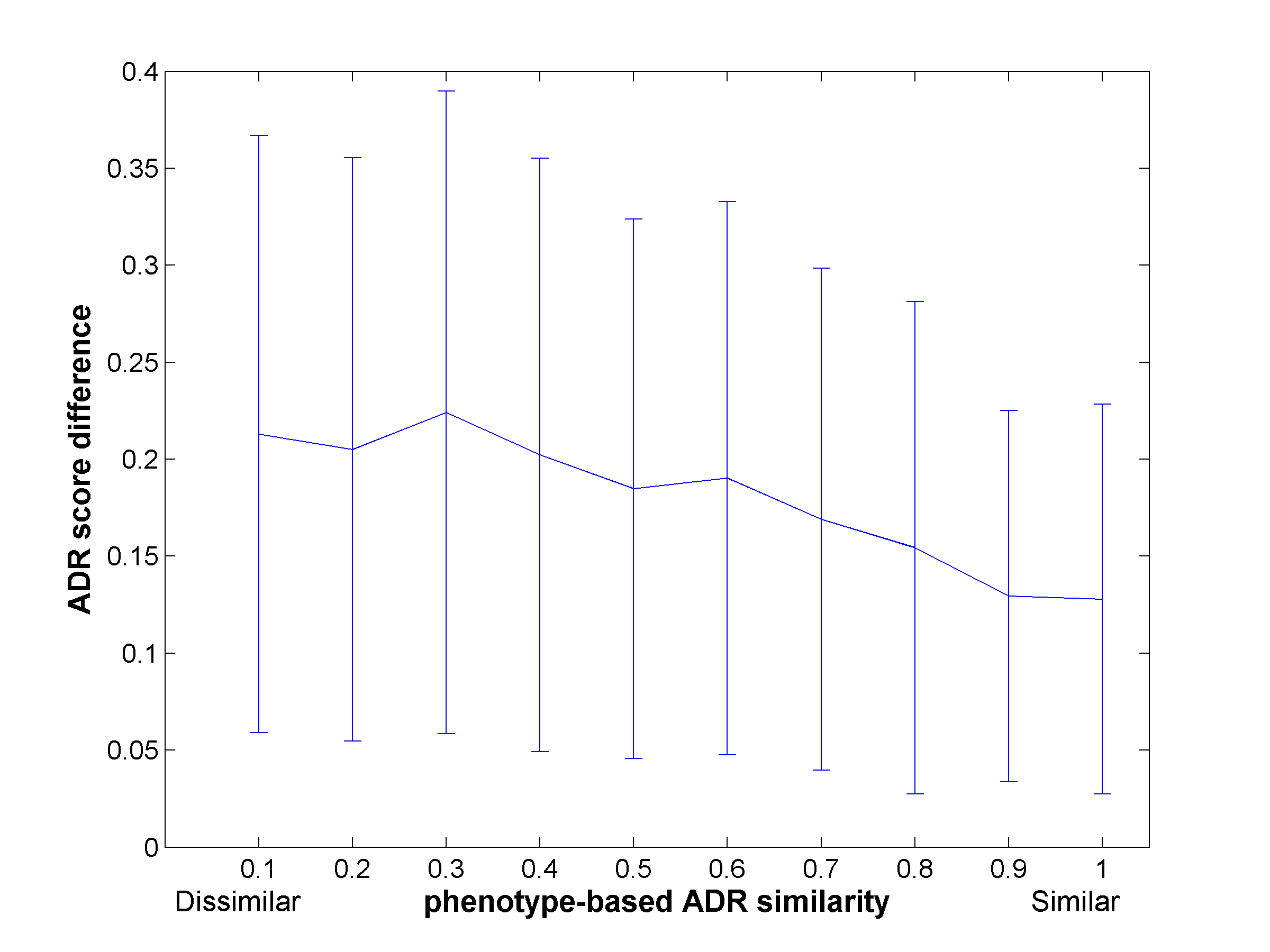

Supplement: Supplementary file 5 [file jmir_v17i3e80_app5.png]
