# Supplementary material for: Ranking Adverse Drug Reactions With Crowdsourcing
Source: J Med Internet Res. 2015 Mar 23;17(3):e80. doi: 10.2196/jmir.3962 (PMC4387295; doi:10.2196/jmir.3962)
Supplement: Supplementary file 3 [file jmir_v17i3e80_app3.pdf]

Supplementary Methods and Legends

Ranking adverse drug reactions with crowdsourcing

Assaf Gottlieb[1]*, PhD; Robert Hoehndorf[2], PhD; Michel Dumontier[3], PhD; Russ B. Altman[1,4], MD, PhD

[1] Department of Genetics, Stanford University, Stanford, California, USA.

[2] Computer, Electrical and Mathematical Sciences & Engineering Division, King Abdullah University of Science and Technology, Thuwal, Kingdom of Saudi Arabia.

[3] Stanford Center for Biomedical Informatics Research, Stanford University, Stanford, California, USA.

[4] Department of Bioengineering, Stanford University, Stanford, California, USA.

Methods

Comparing ADRs using MTurk

We assigned ADR pairwise comparison tasks to MTurk workers. In construction of the pairwise comparisons, we took the following measures in order to maximize the tested pairs and reduce as much as possible potential biases:

Batch construction*:* In order to assess and use intermediate results, we divided the pairwise comparisons to four sets (batches). The batches were completed on different weekdays over a period of a month. We prepared two types of batches: A quality control (QC) randomized batch intended to assess reproducibility of the ranking (14,645 pairs) and controlled randomized (CR) batches. The release of QC batches was intertwined between the CR batches, i.e. the sequence of batch releases to MTurk workers consists of the QC batch, followed by a CR batch (15,721 pairs), the QC batch, a CR batch (29,179 pairs) and the QC batch.).

The QC batch was completed three times in order to check reproducibility of the results and in order to facilitate the construction of the CR batches. It was constructed in a way that maximized the number of pairs that can be tested for triangular inequality (i.e. A vs. B, B vs. C and A vs. C): We randomized the ADR list and used a sliding window such that each ADR was compared to five ADRs preceding it and five ADRs following it in a circular way (total of ten comparisons per ADR or 29,290/2=14,645 pairs overall).

Each CR batch was randomized in such a way that would better utilize the workers. Using an initial crude ranking computed from the first QC batch, we reduced the number of compared pairs that were regarded too easy (involving a severe and a mild ADRs) or regarded as equivalent (ADRs with very close ranks). Equivalent ADRs are typically very similar or ADRs which are hard to compare (i.e. very different in nature, e.g. amnesia vs. hemorrhage) and have the potential to frustrate the MTurk workers in being forced to choose. The way the two CR batches were formed is by binning the crudely-ranked ADRs to ten equally-spaced bins and comparing each ADR to randomly selected ADR from bins that are not too close (adjacent or same bin) and not too far (bins distant up to four bins from

each side of the current bin). The first CR batch totaled in 15,721 pairs compared and the second CR batch in 29,179 pairs. Each batch was completed in approximately two to four hours, totaling in fifteen hours.

Task generation*:* The compared pairs in each batch were divided to worker tasks, each comprising of ten ADR pairwise comparisons. By dividing the entire set of pairs in each batch into five parts, we enabled each MTurk worker to perform between one and five such tasks (10-50 comparisons). Figure S1 shows that most workers compared 10 - 50 pairs of ADRs (i.e. participated in one batch). Each comparison task comprised of ten pairs to compare (See Multimedia Appendix 2 for an example). The user interface provided clickable links to Google queries with the ADR name in order to aid workers learn about ADRs expressed in medical terminology with which they were not familiar. In order to identify reliable workers, each worker task of ten pairs included three pre-defined quality control pairs (and seven randomly chosen pairs from the batch-generated pairs). These quality control pairs were constructed by pairing all combinations from a manually selected set of severe ADRs and a set of mild ADRs: in the QC batches we used sixteen severe and sixteen mild ADRs, resulting in 256 quality control pairs and following the initial crude ranking of the first QC batch, we increased this number to 676 quality control pairs in the CR batches. In order to minimize biases, the location of each pair within the ten pairs in each task and the order of the two compared ADRs in each pair were randomized (including the pre-defined quality control pairs). In order to provide explanation of the ADRs and their medical terminology, we included a link adjacent to every ADR, querying the ADR in Google.

Workers filtering and statistics*:* The workers were required to possess satisfactory task completion record, rejected in less than 5% of past tasks (95% approval rate) and be located in the United States, as a proxy to English proficiency. Using the unique worker identifier across tasks, we were able to filter tasks of unreliable workers who made incorrect choices on more than 20% of the quality control pairs (3% of the 2,589 workers were filtered). Each task, comprised of ten pairwise comparisons, took five minutes to complete on average, yielding 0.45 cents per worker (half a dollar including Amazon's fee). The entire ranking totaled in 146 person days at a cost of $6,300.

Ranking ADRs

In order to rank the ADRs based on the pairwise comparisons, we used linear programming that attempted to retain as much of the original rankings of the workers (in the minimization of the utility function) while ensuring that the ADRs obey the triangular inequality - i.e. for each ADR triplet A, B and C we denote more severe as "greater than", so if A>B and B>C then it follows that A>C. The linear programming optimization function is formulated as:

$$minimize \sum_{i=0}^{n} \sum_{j=0,i\neq j}^{n} W_{ij} \cdot X_{ij} \qquad .1$$

Where n =2,929 is the total number of ADRs and the variable $X_{ij} \in [0,1]$ is the fraction of the time ADR i is more severe than j. W_{ij} are the weights for each pair of ADRs. If the pair of ADRs i and j were compared by MTurk workers, W_{ij} reflects this knowledge by assigning

weight equal to one minus the fraction of times *i* was selected as more severe than *j*, i.e. $W_{ij} = 0, W_{ji} = 1$ if all workers determined that *i* is more severe than *j*. Thus, it places a penalty for choosing $X_{ij} < 1 \; or \; X_{ji} > 0$. Untested pairs receive a uniform weight of 0.5.

The set of constraints are:

$$0 \leq X_{ij} \leq 1 \; \forall_{i \neq j} \qquad .2$$

$$X_{ij} + X_{ji} = 1 \; \forall_{i \neq j} \text{ (no inconsistencies)} \qquad .3$$

$$X_{ij} \leq X_{ik} + X_{kj} \; \forall_{i \neq j \neq k} \text{ (triangular inequality)} \qquad .4$$

Finally, the score of each ADR *i* is a "Borda count" [1], summing over all its pairwise ranks X*ij*

$$Score(i) = \sum_{j=0, i \neq j}^{n} X_{ij} \qquad .5$$

As the number of variables in this linear programming scheme is quadratic with *n* (all pairs) and the constraints involving triangular inequalities is cubic with *n*, we found it infeasible to solve for n=2,929 in terms of time and space. We therefore created 8,787 samples of size 100 (three samples per ADR), ranked each sample independently and finally created a global score for all the ADRs. In order to make each sample dense with inter-ADR comparisons, we created an iterative process whereby starting with each ADR *i*, we iteratively add the ADRs that were compared to *i* (first tier), the ADRs compared to those in the first tier and so on, until reaching n=100. In case the last tier brought the number to more than a hundred, we chose randomly from the last tier in order to reach a 100 (totaling in three random samples per ADR). Each ADR appeared in 300±87 samples and on average shared at least one sample with 2,925±4 (99.9% ±0.1%) of the other ADRs. Arranging all the samples in a fixed size has the advantage of constructing the constraints only once, since the utility function (equation 1) is the only part in the linear programming that depends on the worker comparison results. Computation of each sample took 58 seconds on average. Finally, the ranking of an ADR *i* is the average ranking across all the samples it participated in. Figure S2 shows that we obtain a stable ranking (Spearman correlation, ρ= 0.97) when the number of ranked samples exceeds 1000 samples. The linear programming was implemented in MATLAB using IBM CPLEX package version 12.6 [2].

Optimizing equivalence classes

Using independent rankings computed from each of the repeated batches, we obtained the standard deviation per ADR rank. Based on these standard deviations, we selected ADR equivalence classes using balanced one-sided analysis of variance (ANOVA). In order to partition the ADRs to classes, we used a two-step greedy algorithm. In the first step, we divided the ADRs into k-equal sized bins and selected k with minimal p-value (k=12). For the next step, we merged the twelve equal-sized bins to six classes by randomly selecting and merging adjacent classes and computing their ANOVA p-value. We tested both a gradient descent approach and a simulated annealing approach. In the gradient descent approach, we merged classes if the resulting p-value was lower than before the merge. The gradient-descent approach was iterated a 1000 times to avoid local minima. The simulated

annealing approach was performed with a 1000 iterations. Both methods converged to the same final six clusters with minimal ANOVA p-value.

Computing sematic similarity for ADRs

The semantic similarity between two ADRs is computed based on the hierarchical structure of HPO. As an example, vocal cord paresis a similar semantic meaning as one of its symptoms – hoarseness. Following the method proposed by [3], we computed the information content of each concept c in the hierarchy as

$$IC(c) = -\frac{log\left(\frac{|leaves(c)|}{|\text{subsumers}(c)|} + 1\right)}{(\text{max\_leaves} + 1)} \quad .6$$

, where leaves(c) is the subset of concepts that are a specialization of c (below c in the hierarchy) and do not have a sub-concept in HPO, and subsumers(c) is the subset of concepts that c specializes (including c itself). This ratio is normalized by the total number of leaves of the root concept (max_leaves). The information content measure produces values in the range of 0 and 1.

Similarity between two concepts $c1$ and $c2$ in HPO with the most informative common ancestor m is computed using the measure of [4]:

$$similarity\ (c1, c2) = 1 - (IC(c1) + IC(c2) - 2IC(m)) \quad .7$$

We combined the 3,730 mappings between HPO concepts and unified medical language system (UMLS) [5] concepts made available by HPO with 197 new HPO to UMLS mappings using an exact string matched to the UMLS concepts available from SIDER2. Finally, 793 ADRs (out of the 2,929 ADRs) were mapped to unique HPO terms.

Abbreviations

ADR: adverse drug reaction
ANOVA: analysis of variance
CR: controlled randomized
FDA: Food and Drug Administration
HPO: Human Phenotype Ontology
MTurk: Amazon Mechanical Turk
QC: quality control
UMLS: unified medical language system

Supplementary Figures

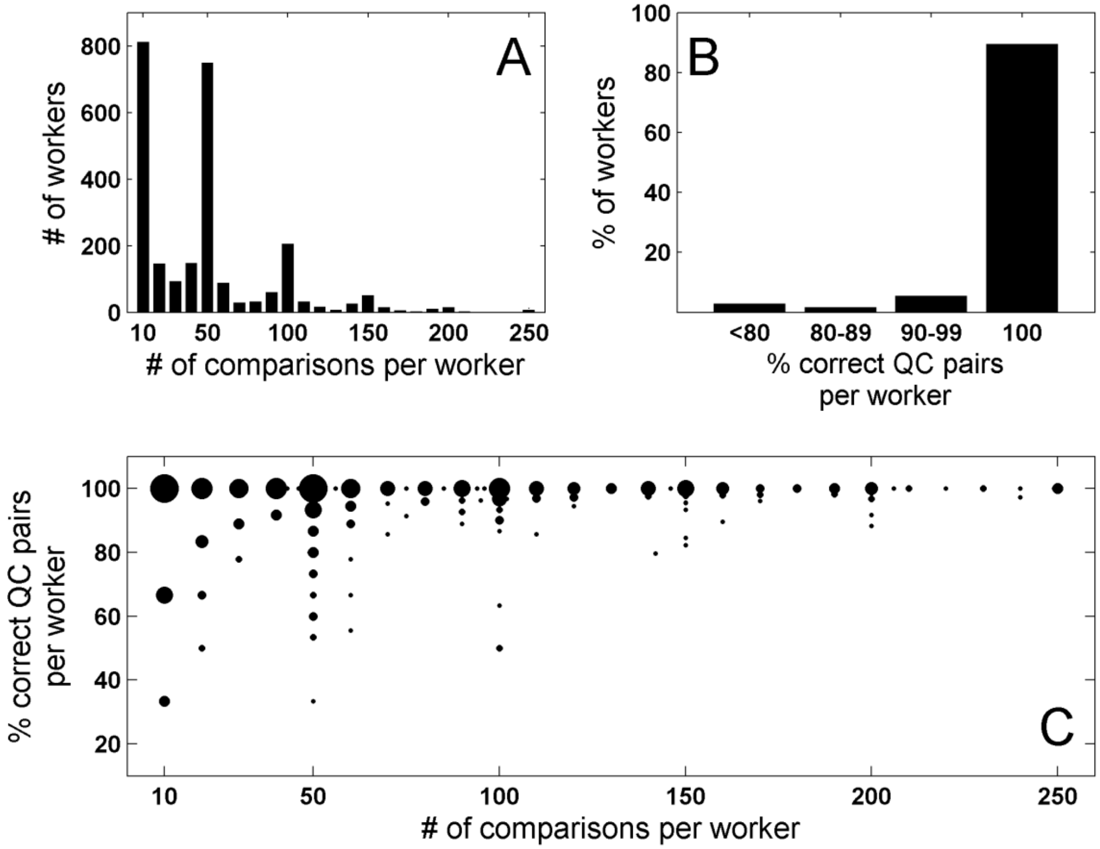

Figure S1. MTurk worker statistics. Displayed are (A) the number of comparisons per worker (a worker was allowed to do 10-50 comparisons in each batch), (B) the percent of correct answers on quality control (QC) comparisons and (C) the percent of correct answers on quality control (QC) comparisons as a function of the # of comparisons a worker did. Point size is proportional to the number of workers (larger=more workers).

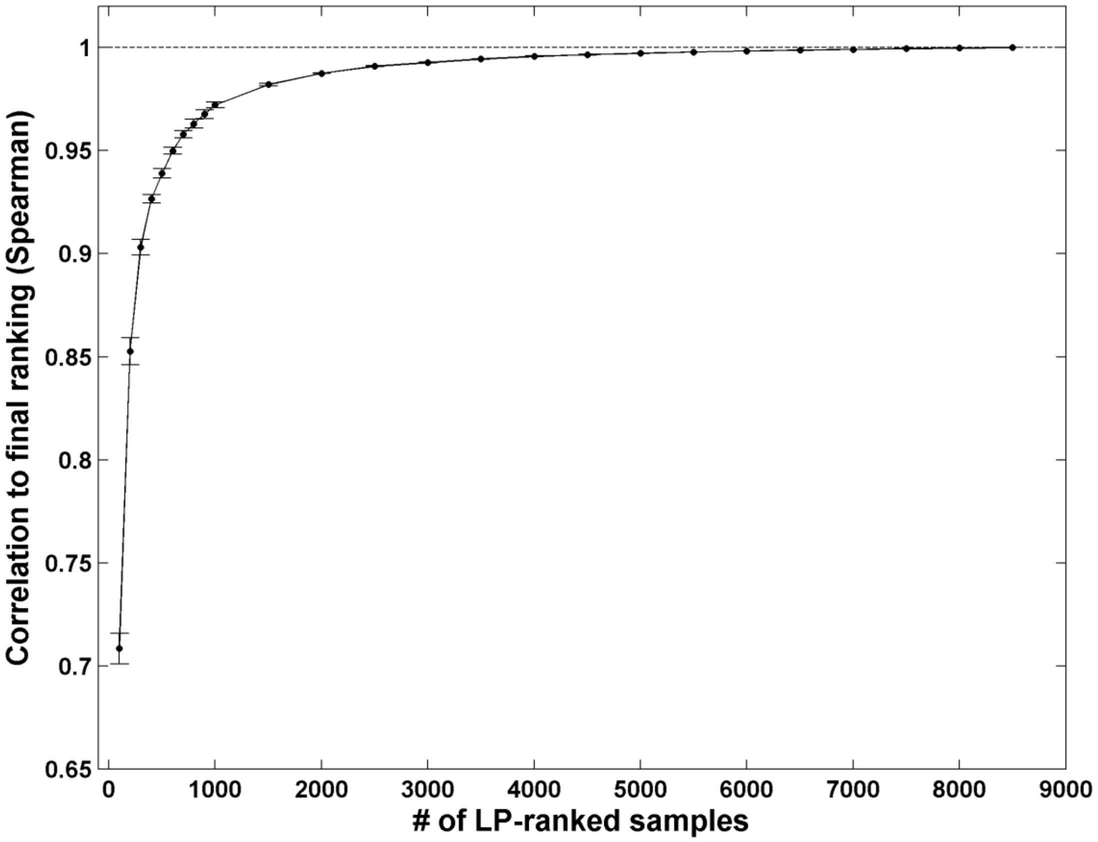

Figure S2. The Spearman correlation between the final ADR ranking and intermediate rankings, as a function of the number of samples used to compute the intermediate rankings.

Multimedia Appendix Legends

Multimedia Appendix 1. An example of a comparison presented to an MTurk worker.

Multimedia Appendix 2.(Table S1). The MTurk workers pairwise comparisons used to compute the ranking.

Multimedia Appendix 4. (Table S2). Ranked list of ADRs with their reported frequency.

Multimedia Appendix υ. The correlation between ADR semantic similarity and the mean difference in severity scores, computed for 793 ADRs.

Multimedia Appendix 6. (Table S3). Top prescribed drug in 2013 that have novel severe ADRs in OFFSIDES database.

Multimedia Appendix 7. (Table S4). Genes and their most severe associated ADRs.

References

1. Dym, C.L., Wood, W.H. & Scott, M.J. Rank ordering engineering designs: pairwise comparison charts and Borda counts. *Research in Engineering Design* **13**, 236-242 (2002).
2. CPLEX, I.I. (IBM, 2010).
3. Sánchez, D., Batet, M. & Isern, D. Ontology-based information content computation. *Knowledge-Based Systems* **24**, 297-303 (2011).
4. Jiang, J.J. & Conrath, D.W. Semantic similarity based on corpus statistics and lexical taxonomy. *arXiv preprint cmp-lg/9709008* (1997).
5. Bodenreider, O. The unified medical language system (UMLS): integrating biomedical terminology. *Nucleic acids research* **32**, D267-D270 (2004).
